# Structural insights into how augmin augments the mitotic spindle

**DOI:** 10.1038/s41467-023-37625-3

**Published:** 2023-04-13

**Authors:** Szymon W. Manka

**Affiliations:** MRC Prion Unit at UCL, Institute of Prion Diseases, University College London, 33 Cleveland Street, London, W1W 7FF UK

**Keywords:** Mitotic spindle, Cytoskeletal proteins, Cryoelectron microscopy, Microtubules

## Abstract

Cell division critically requires amplification of microtubules (MTs) in the bipolar mitotic spindle. This relies on the filamentous augmin complex that enables MT branching. Studies by Gabel et al., Zupa et al. and Travis et al. describe consistent integrated atomic models of the extraordinarily flexible augmin complex. Their work prompts the question: what is this flexibility really needed for?

## The role of augmin

In metazoa, every cell division is preceded by an assembly of the karyokinetic spindle, a tightly regulated dynamic MT-based micromachine. Spindle MTs drive faithful segregation of duplicated chromosomes (sister chromatids), to maintain genome integrity of dividing cells. Inaccurate segregation of chromosomes, if not immediately fatal, causes aneuploidy that can lead to cancer (vast majority of tumours contain cells with extra or missing chromosomes). Fulfilment of the fundamentally important task of accurate chromosome segregation is, therefore, safeguarded by three pathways of spindle MT nucleation: a) an overarching centrosomal nucleation, which originates from two microtubule organising centres (MTOCs), located at opposing ends of the cell periphery that become spindle poles, b) chromatin-mediated nucleation at the equator (spindle centre), where chromosomes line up, and c) intra-spindle, branching nucleation, responsible for the bulk of spindle MTs. This latter MT branching pathway depends on a conserved protein complex called augmin^1^.

Augmin deficiencies show severe spindle defects, mitotic delay, and massive apoptosis, while conditional knockout of HAUS6 in apical progenitors of the developing mouse brain is embryonically lethal^2^. Besides its role in spindle assembly, augmin boosts MT density in neuronal dendrites and ensures uniform MT polarity in axons.

Augmin is a hetero-octamer of homologous to augmin subunits (HAUS)1-8, grouped into two stable tetrameric segments, TII and TIII, serving distinct functions. TIII mediates recruitment of the principal MT nucleator ɣ-TuRC (ɣ-tubulin ring complex, the same as in MTOCs) via the adaptor protein NEDD1 (neural precursor cell expressed developmentally down-regulated protein 1). TII mediates attachment to lateral surfaces of pre-existing MTs, thereby installing MT branching templates at a shallow angle, for robust, polarity-controlled spindle augmentation^3,4^. The ensuing dense MT array reaches the equator for efficient capture of sister chromatids.

## Determining the structure of the augmin complex

Recent in vitro reconstitution studies using native or recombinant purified components of the minimal MT branching machinery from fruit fly (*D. melanogaster*), frog (*X. laevis*) or human^3–7^ brought us closer to answering the long-standing question of how branching MT nucleation occurs. Despite these advances, the three-dimensional structure of augmin remained elusive, hindering detailed mechanistic understanding of the augmin function. It has been particularly puzzling how the extremely flexible augmin scaffold^4^ would be able to maintain a defined spindle polarity.

In their elegant work, Gabel et al., Zupa et al. and Travis et al. elucidate the molecular architecture and conformational plasticity of the augmin holocomplex from human^8^ and *X. laevis*^9,10^. They integrated Artificial Intelligence-based structure prediction (ColabFold^11^ or AlphaFold-Multimer^12^) with complimentary structural biology techniques, such as single-particle cryogenic electron microscopy (cryo-EM) and cross-linking mass spectrometry, or more classic model validation using bulky tags visualised by negative stain EM. Notably, it was not possible to delineate the inter-connected structural arrangement of the HAUS subunits in the augmin holocomplex by any of these methods alone.

**Fig. 1:**
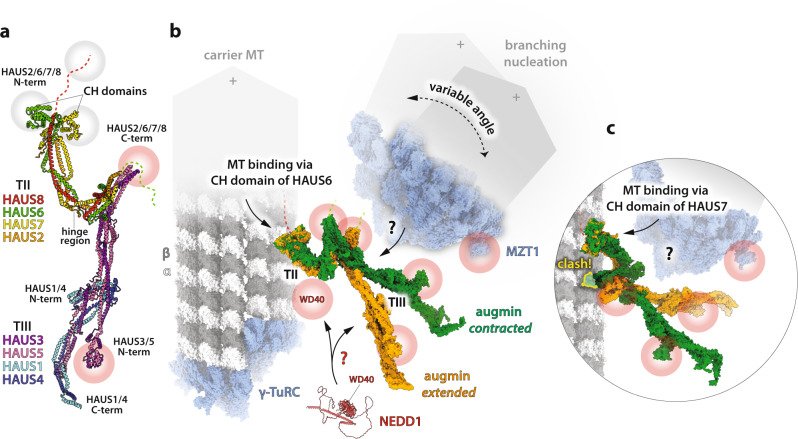
Possible scenarios for augmin-mediated MT branching. **a** Atomic model of the augmin complex (*extended* conformation) by Gabel et al. (pdb code: 7SQK). Red and green dashed lines: disordered N- and C-terminal domains of HAUS8 and 6, respectively. Grey halos: MT interaction sites. **b** Depiction of MT branching machinery and its inherent flexibility based on augmin binding to pre-existing (carrier) MT (α/β-tubulin dimer, pdb code: 6REV) via the CH domain of HAUS6. Stable MT minus-end is anchored in γ-TuRC (pdb code: 7QJ5), while the dynamic plus-end (+) is indicated with arrowheads. *Contracted* augmin model (green) by Zupa et al. (pdb code: 8AT3), *extended* augmin model (orange) as in **a**. Red halos: putative NEDD1 interaction sites. NEDD1 model: AlphaFold2 prediction (low-confidence, except for the WD40 β-propeller domain); shown in scale to indicate spatial span—several monomers/oligomers may be involved. **c** Augmin binding to carrier MT via the CH domain of HAUS7 is not compatible with the current *contracted* augmin model (results in a severe clash with the carrier MT lattice).

The three independent integrative approaches yielded coherent models of conserved α-helical coiled-coils organised into distinct TII and TIII bundles: HAUS2,6–8 subunits of the U-shaped TII are connected via their C-termini to the stalk-like TIII, composed of HAUS1,3–5 (Fig. 1a). While the core of TIII appears to be rigid, TII exhibits remarkable clamp-like motions, supporting open (*extended*) and closed (*contracted*) conformations (Fig. 1b, c). Strikingly, the N-termini of HAUS6 and 7 assume a globular, calponin homology (CH) domain fold, most similar to that of the MT-binding CH domain of the kinetochore NDC80 complex (Fig. 1a), which strongly suggests a direct role in MT binding (Fig. 1b, c), in line with the existing orthogonal data^3^. In addition, Travis et al. show a comprehensive structure prediction panel that highlights structural conservation of augmin subunits across eukaryotes, despite low sequence similarity in distant orthologs (as in *Drosophila*).

## How the augmin structure explains its function (or not)

The unstructured, positively charged N-terminal tail of HAUS8 (Fig. 1, dashed red line), has previously been reported as critical for targeting augmin to MTs^3^. Critical does not mean sufficient, since MT binding affinity of the HAUS8 N-terminus alone is roughly an order of magnitude weaker than that of the HAUS6-8 dimer^3^, implying a strong synergistic effect of the combined MT binding modes (also reminiscent of the composite MT-binding mode of NDC80). Accordingly, TII can probably be described as a foot that is flexible yet able to firmly position augmin on an existing MT track (target MT lattice). This association occurs via one or both of its CH domains (Fig. 1b, c), following initial weak diffusion along the track, supported by the disordered tail of HAUS8^7^.

MT anchorage via a single CH domain of HAUS6 seems most compatible with the established augmin conformations and with augmin function, as depicted in Fig. 1b. An alternative MT binding mechanism, involving the CH domain of HAUS7 (or both CH domains) is considered in Fig. 1c. Such binding appears incompatible with the currently proposed *contracted* configuration of the augmin complex, which could represent a mechanism for restricting augmin movements. Neither of these two MT binding scenarios can decidedly exclude the possibility of functional γ-TuRC docking via NEDD1 to the base of the TIII stalk, as predicted from the putative NEDD1 interaction sites (Fig. 1b, c, red halos) comprising the partly unstructured C-terminal half of HAUS6 (Fig. 1, dashed green line)^3^ and the N-terminal HAUS3/5 bundle^10^. In other words, either of the two MT binding modes appears plausible, however, they seem mutually exclusive from the structure–function point of view. Probably only one of them is utilised by the cell, unless NEDD1 binding, and/or another MT branching stimulator or modulator (such as TPX2^13^), can somehow functionally reconcile both MT binding modes. Of note, AlphaFold2 models suggest that the intrinsic conformational flexibility of augmin might exceed the range of conformations observed by cryo-EM imaging of augmin particles attached to a continuous carbon film, as was the case in all three studies. Consequently, harnessing augmin’s flexibility in the context of the MT branching process may depend on additional factors such as augmin oligomerisation^7^ or NEDD1 binding to the MT lattice via its WD40 β-propeller domain (Fig. 1b), which may act to restrict MT branching angles^7^.

In conclusion, one of the key remaining challenges to unravel some of the remaining mysteries and ambiguities of the MT branching process is to establish how NEDD1 bridges γ-TuRC—or the MZT1 sub-complex therein^7^ (Fig. 1b)—with augmin, and how the whole machinery interacts with the MT track.

## Could augmin’s flexible foot have wider functional relevance?

The role of augmin’s flexibility and the TII hinge in particular is intriguing. It may represent yet another redundancy in ensuring that all possible kinetochore locations are explored, but it is easy to envisage adequate enhancement of MT density within the spindle also by a more rigid MT branching mechanism. Perhaps the role of the flexible TII foot is not limited to permitting a certain range of MT branching angles, but is primarily to endow the spindle with a certain shock-absorbing capacity, thereby rendering it more elastic (and less brittle) in response to various external stresses. In other words, spindles may be built for cellular ‘earthquakes’.

It is not certain whether the clamp-like TII module is to any degree elastic (tending to regain a particular conformation after removal of external load) or just plastic (readily retaining alternative conformation(s)), but perhaps it can help to dampen and/or distribute the impact of an external force, exerted for instance by flowing blood on proliferating endothelial cells at sites where blood vessel lining is damaged. Such an adaptive force-dampening mechanism might also benefit layers of regenerating or dividing cells in internal organs or skin, which are subjected to various mechanical stresses.

Alternatively, the flexible foot of augmin might have a role in sensing and/or transducing mechanical signals to direct (or mould) mitotic spindle formation in accordance with external cues. Such a role would align well with the existing evidence that various mechanical forces help orient the mitotic spindle in different tissues. In any case, characterising the relevance of augmin’s flexibility in the context of spindle assembly remains an exciting endeavour for future research.
